# Large corneal epithelial detachment as a complication of wound burping to release aqueous humor for elevated intraocular pressure following cataract surgery

**DOI:** 10.1093/jscr/rjae244

**Published:** 2024-06-22

**Authors:** Jarryl H J Tsai, Jonathan T W Au Eong, Kah-Guan Au Eong

**Affiliations:** Lee Kong Chian School of Medicine, Nanyang Technological University, 11 Mandalay Road, Singapore 308232, Singapore; Lee Kong Chian School of Medicine, Nanyang Technological University, 11 Mandalay Road, Singapore 308232, Singapore; Lee Kong Chian School of Medicine, Nanyang Technological University, 11 Mandalay Road, Singapore 308232, Singapore; International Eye Cataract Retina Center, Mount Elizabeth Medical Center and Farrer Park Medical Center, 1 Farrer Park Station Road #14-07/08, Connexion, Singapore 217562, Singapore; Department of Ophthalmology and Visual Sciences, Khoo Teck Puat Hospital, 90 Yishun Central, Singapore 768828, Singapore

**Keywords:** aqueous humor release, wound burping, cataract surgery

## Abstract

Wound burping is a technique used to treat intraocular pressure spikes in the immediate postoperative period after cataract surgery. A 55-year-old man with no history of glaucoma presented with painless blurring of vision in his left eye following cataract surgery 20 days earlier. Ophthalmic examination disclosed elevated intraocular pressure, mild conjunctival hyperemia, corneal microcystic epithelial edema, and mild anterior chamber reaction. In an attempt to lower the intraocular pressure quickly, the corneal wound was ‘burped’ at the slitlamp. Upon burping the wound, a large epithelial bulla formed instantly in the cornea. The patient’s blinking caused the corneal epithelial bulla to burst and collapse. Examination the next day disclosed the detached epithelium had sloughed off completely. The epithelial defect healed gradually over 10 days. Wound burping to release aqueous humor after the corneal epithelium has healed over the surgical incision can result in corneal epithelial detachment and should be avoided.

## Introduction

Aqueous humor release, commonly known as wound burping, is a technique which has been used for several decades by surgeons to treat intraocular pressure (IOP) spikes in the immediate postoperative period following cataract surgery in the phacoemulsification era. It is done by applying a small amount of pressure posterior to the corneal incision, thus reopening the surgical wound and allowing aqueous humor out of the anterior chamber. Despite its popularity, there is no published literature on the indications, efficacy, and safety of this technique. We report a case of large corneal epithelial detachment following wound burping after cataract surgery to highlight this potential complication.

## Case report

A 55-year-old man presented with painless blurring of vision in his left eye following phacoemulsification and monofocal intraocular lens implantation 20 days earlier by another ophthalmologist. The surgery was performed through a superonasal 2.75-mm clear cornea incision. He had a mild epiretinal membrane in his left eye but did not have any history of glaucoma.

**Figure 1:**
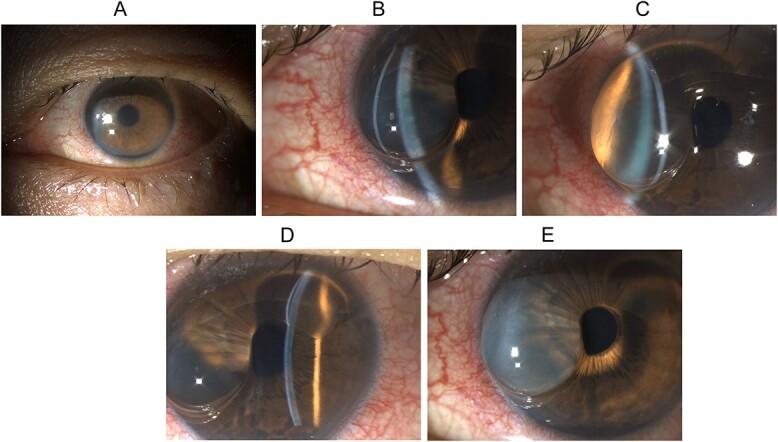
Slit lamp biomicroscopy of the left eye on presentation and upon wound burping: (A) diffuse illumination of the anterior segment showing conjunctival hyperemia, diffuse corneal haze, and microcystic epithelial edema; (B) direct focal illumination using a slit beam coming from the examiner’s left side showing separation of the corneal epithelium from the underlying corneal stroma in the nasal half of the cornea; (C) direct focal illumination using a slit beam coming from the examiner’s right side showing the huge corneal epithelial detachment in the nasal half of the cornea, and the detached epithelium is relatively clear in the first minute after the wound burping, and (D) direct focal illumination using a slit beam coming from the examiner’s left side showing separation of the corneal epithelium from the underlying corneal stroma in the superior quarter of the cornea; the C-shaped corneal epithelial detachment is clearly seen, and (E) diffuse illumination of the anterior segment showing the detached corneal epithelium had turned hazy in the second minute after the wound burping.

His uncorrected visual acuity was 20/70 correctable with pinhole to 20/50 in the left eye. The IOP was 34.1 mmHg. Ophthalmic examination disclosed mild conjunctival hyperemia, corneal haze, and microcystic epithelial edema (Fig. 1A). The anterior chamber was deep with mild anterior chamber reaction. There was no forward displacement of the IOL-iris diaphragm.

In an attempt to lower the IOP quickly, the corneal wound was ‘burped’ with a sterile cotton bud under topical anesthesia using aseptic techniques. He was also given topical and oral antiglaucoma medications.

Upon burping the wound, a large relatively clear C-shaped epithelial bulla formed instantly in the nasal half (Fig. 1B and C) and superior quarter of the cornea (Fig. 1D). After 1 min, the detached corneal epithelium gradually became hazy (Fig. 1E) although its size remained stationary.

**Figure 2:**
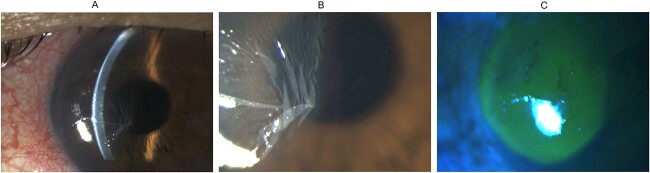
Slit lamp biomicroscopy and fluorescein staining of the left eye after bursting of corneal epithelial bulla: (A) direct focal illumination using a slit beam coming from the examiner’s left side showing the corneal epithelium resting on the underlying corneal stroma in the nasal half of the cornea after the corneal epithelial bulla had burst; (B) diffuse illumination of the anterior segment showing redundant folds of corneal epithelium after the epithelial bulla had burst; (C) cobalt blue light examination after instillation of fluorescein sodium showing fluorescein staining of the deflated bulla.

The patient’s blinking caused the corneal epithelial bulla to burst and leak in the second minute, resulting in its collapse (Fig. 2A). The detached corneal epithelium formed redundant folds after the bulla flattened (Fig. 2B). Upon instillation of fluorescein sodium, the dye entered and stained the deflated bulla (Fig. 2C).

The patient developed left ocular pain in the night and examination the next day disclosed the detached epithelium had sloughed off completely, resulting in a large corneal epithelial defect. Several Descemet’s folds were also noted and the IOP had reduced to 18.4 mmHg.

He was treated with copious preservative-free sodium hyaluronate 0.3% eyedrops and the epithelial defect healed gradually over 10 days. The IOP remained normal at all subsequent visits and the antiglaucoma medications were gradually reduced and finally discontinued after 43 days. The Descemet’s folds resolved over a few months and the best-corrected visual acuity improved to 20/25 after 4 months. The final visual acuity was limited by the pre-existing epiretinal membrane in the left eye.

## Discussion

To the best of our knowledge, this is the first report of corneal epithelial detachment occurring as a complication of wound burping to release aqueous humor for elevated IOP following cataract surgery.

Wound burping is usually a low-risk procedure to relieve acute IOP elevation in postphacoemulsification patients during the early postoperative period when the corneal epithelium has not fully healed over the surgical incision. This healing process takes about 7–14 days [1]. Even in cases when the corneal epithelium has newly formed, healing is not considered complete until a week later when permanent anchoring units are formed [2]. Before that, pressure on the posterior wound lip and the rapid egress of aqueous humor tend to break the newly healed epithelium and reopen the wound.

In our patient, the complication arose because the procedure was performed 20 days after the cataract surgery when the corneal epithelium had healed over the corneal incision and pressure on the posterior wound lip reopened the subepithelial corneal layers without breaking the corneal epithelium. The aqueous humor exiting rapidly through the subepithelial corneal layers failed to rupture the epithelium. Instead, it dissected the subepithelial plane to detach a sizeable area of the intact corneal epithelium.

To prevent this complication, slit lamp examination using cobalt blue light and fluorescein can help to determine if the corneal epithelium has healed [3]. Complete healing of corneal epithelium is a relative contraindication to burping and other IOP-lowering methods such as pharmacological treatment [4] and anterior chamber paracentesis using a needle [5] should be considered.

## Conclusions

In summary, wound burping to release aqueous humor after the corneal epithelium has healed over the surgical incision can result in detachment and sloughing of the corneal epithelium and should be avoided.
